# Manual and Automatic Image Analysis Segmentation Methods for Blood Flow Studies in Microchannels

**DOI:** 10.3390/mi12030317

**Published:** 2021-03-18

**Authors:** Violeta Carvalho, Inês M. Gonçalves, Andrews Souza, Maria S. Souza, David Bento, João E. Ribeiro, Rui Lima, Diana Pinho

**Affiliations:** 1 Mechanical Engineering and Resource Sustainability Center (MEtRICs), Mechanical Engineering Department, University of Minho, 4800-058 Guimarães, Portugal; violeta.carvalho@dem.uminho.pt (V.C.); diana.pinho@inl.int (D.P.); 2 Instituto Superior Técnico, Universidade de Lisboa, Av. Rovisco Pais, 1049-001 Lisboa, Portugal; inesmaiag@gmail.com; 3 Centro para a Valorização de Resíduos (CVR), University of Minho, 4800-028 Guimarães, Portugal; andrewsv81@gmail.com; 4 Center for MicroElectromechanical Systems (CMEMS), University of Minho, 4800-058 Guimarães, Portugal; sabrinasouza680@gmail.com; 5 Transport Phenomena Research Center (CEFT), Faculdade de Engenharia da Universidade do Porto (FEUP), Rua Dr. Roberto Frias, 4200-465 Porto, Portugal; davidbento@ipb.pt; 6 Polytechnic Institute of Bragança, ESTiG/IPB, C. Sta. Apolónia, 5300-857 Bragança, Portugal; jribeiro@ipb.pt; 7 Centro de Investigação de Montanha (CIMO), Polytechnic Institute of Bragança, 5300-252, Bragança, Portugal

**Keywords:** blood flow, particle tracking, red blood cells, manual methods, automatic methods, image analysis, biomicrofluidics

## Abstract

In blood flow studies, image analysis plays an extremely important role to examine raw data obtained by high-speed video microscopy systems. This work shows different ways to process the images which contain various blood phenomena happening in microfluidic devices and in microcirculation. For this purpose, the current methods used for tracking red blood cells (RBCs) flowing through a glass capillary and techniques to measure the cell-free layer thickness in different kinds of microchannels will be presented. Most of the past blood flow experimental data have been collected and analyzed by means of manual methods, that can be extremely reliable, but they are highly time-consuming, user-intensive, repetitive, and the results can be subjective to user-induced errors. For this reason, it is crucial to develop image analysis methods able to obtain the data automatically. Concerning automatic image analysis methods for individual RBCs tracking and to measure the well known microfluidic phenomena cell-free layer, two developed methods are presented and discussed in order to demonstrate their feasibility to obtain accurate data acquisition in such studies. Additionally, a comparison analysis between manual and automatic methods was performed.

## 1. Introduction

Blood flow in microcirculation is crucial for the normal function of tissues and organs. Therefore, a detailed study of blood flow patterns and blood cells flowing in microvessels, microchannels and organs-on-chip is essential to provide a better understanding of the blood rheological properties and disorders in microcirculation [1,2,3,4,5,6,7]. One of the first techniques used for the study of flow patterns was the phase-contrast magnetic resonance imaging (PC-MRI). However, the technique requires long acquisition times and has low resolution [8,9]. Other techniques have been developed and combined to improve the acquisition and image processing. One of the most reliable ways to measure velocity fields in microcirculation is using Eulerian methods, such as the conventional micro-particle image velocimetry (PIV) [1,6,10,11,12] or the confocal micro-PIV [1,2,6,13]. The micro-PIV technique is one of the best suitable methodologies to study blood flow phenomena in microcirculation. Some studies have also combined PIV with ultrasounds (Echo-PIV) [14,15]. However, most in vivo measurements contain physiological fluids with high concentrations of blood cells and as a result, the amount of tracer particles captured within the fluid is often very low [5]. Other approaches for blood flow studies are particle illumination photography, laser doppler velocimetry, fluorescent cytometry [16,17] and computer fluid dynamics [17,18].

In microcirculation, the study of red blood cells (RBCs) flowing in microvessels and microchannels and the study of the cell-free layer (CFL) thickness in different microchannels geometries are very important to get a better understanding of the blood rheological properties and disorders in microvessels in a fast and accurate way. The presence and physiological characteristics of other cell types are also of great clinical relevance [19]. In this kind of study, the image analysis has an important role to obtain crucial information about blood rheology. For blood flow in microvessels, where there is a large number of interacting cells, manual tracking methods have been used to accurately track individual deformable cells flowing through glass capillaries [1,11,20], straight polydimethylsiloxane microchannels [21], stenotic arteries [22,23], hyperbolic contractions [24], and bifurcations [25]. However, the manual data collection is extremely time-consuming to have a statistically representative number of samples and may introduce operators’ errors that eventually limit the application of these methods many times at different conditions [26]. Hence, it is crucial to develop versatile and automatic methods able to automatically track and compute multiple cell trajectories and able to measure the cell-free layer thickness in a network of microchannels.

The purpose of this work is to review the state of the art of techniques used in in vitro blood flow studies and two developed methods (i) an automatic method to track RBCs flowing through microchannels and (ii) an automatic method to measure the CFL thickness in microchannels with bifurcations and confluences will be present and discuss.

This work is organized as follows, firstly an overview of methods used over the last years in the study of blood cells’ morphology and tracking in in vitro blood flows is described. Secondly, a brief introduction to ImageJ, the image analysis software used to obtain manual data, will be made. Then, in Section 4, the results of manual and automatic methods applied were demonstrated and are discussed by the comparison with the manual data. Finally, a conclusion and future directions for the present work were discussed in Section 5.

## 2. An Overview of Image Analysis Methods for Microfluidic Blood Phenomena Quantification

### 2.1. Image Segmentation and Thresholding 

Image analysis processing is a vast area that provides a large number of viable applications that can involve some steps such as image acquisition, image preprocessing, image segmentation, image post-processing and image analysis. Image segmentation is one of the most important and critical elements in automated image analysis, which consists in dividing a digital image into multiple regions, based on a set of pixels or objects, to simplify and/or change the representation of an image [27,28,29]. A variety of techniques can be applied: simple methods such as thresholding, or complex methods such as edge/boundary detection or region growing.

The literature contains hundreds of segmentation techniques [30,31], but there is no single method that can be considered good enough for all kinds of images. The main purpose of segmentation is to divide an image into regions of interest with similar gray-levels and textures in each region [32]. Segmentation methods change according to the imaging modality, application domain, method type—automatic or semi-automatic, depending on the image quality and the image artifacts, such as noise. Some segmentation methods may require image preprocessing prior to the segmentation algorithm [33,34]. Databases with algorithms to compensate for the uncertainties present in real-life datasets were developed [35]. On the other hand, some other methods apply post-processing to overcome the problems arising from over-segmentation. Overall, segmentation methods can be grouped into thresholding, boundary detection, and region growing [27,29,31,36,37]. Those methods vary in the way that the image features are treated and the way the appearance and shape of the target are modeled [38].

Thresholding methods assign pixels with intensities below a certain threshold value into one class and the remaining pixels into another class and form regions by connecting adjacent pixels of the same class, that is, in the thresholding process, each pixel in a grayscale is recognized as either an object or background. The more advanced method creates histograms, oriented to the intensity of grayscale or color, showing the frequency of occurrence of certain intensities in an image so that the regions and objects are recognized from these data [28,29,30]. Thresholding methods work well on simple images where the objects and background have distinctively different intensity distributions. Boundary extraction methods use information about intensity differences between adjacent regions to separate the regions from each other. If the intensities within a region vary gradually but the difference of intensities between adjacent regions remains large, boundary detection methods can successfully delineate the regions [28,29,30,39]. Region growing methods form regions by combining pixels of similar properties [39,40].

### 2.2. Blood Cell Image Segmentation and Tracking

Over the last years, many studies have been conducted in the area of general segmentation methods that can analyze different types of medical images. Most used images are acquired during a diagnostic procedure and useful information is extracted for the medical professional. The development of image analysis in biomedical instrumentation engineering has the purpose of facilitating the acquisition of information useful for diagnosing, monitoring, treating or even investigating certain pathological conditions. It is important to always have in mind that the main purpose of biomedical imaging and image analysis is to provide a certain benefit to the subject or patient [41,42].

In normal human blood microscopic images, a high accumulation of RBCs could be observed, which results in the existence of touch and overlap between these cells [42]. These are two difficult issues in image segmentation where common segmentation algorithms cannot solve this problem [43]. Besides that, staining and illumination inconsistencies also act as uncertainty to the image [44]. This uncertainty makes the blood cell image segmentation a difficult and challenging task [43]. Numerous segmentation methods from peripheral blood or bone marrow smears have been proposed and most of them are region-based or edge-based schemes [42,45].

Jianhua et al. [46] developed an iterative Otsu’s approach based on a circular histogram for the leukocyte segmentation. R. Sukesh Kumar et al. [47] developed two methods of color image segmentation using the RGB space as the standard processing space. These techniques might be used in blood cell image segmentation. Color images are a very rich source of information, because they provide a better description of a scene as compared to grayscale images. Hence, color segmentation becomes a very important and valuable issue [42,47]. For instance, Huang et al. [48] investigated a method based on the Otsu’s method to segment and then recognize the type of leukocyte based on the characteristics of the nucleus. Willenbrock et al. [49] developed a program for image segmentation to detect both moving and stagnated cells in phase-contrast images. The program contributed to the study of the integrin LFA-1 mediation of lymphocyte arrest.

Khoo Boon et al. [50] performed comparisons between nine image segmentation methods which are gray-level thresholding, pattern matching, morphological operators, filtering operators, gradient-in method, edge detection operators, RGB color thresholding, color matching, HSL (hue, saturation, lightness) and color thresholding techniques on RBC. They concluded that there is no single method that can be considered good for RBC segmentation [42,50]. Meng Wang et al. [51] presented segmentation and online learning algorithms in acquiring, tracking and analyzing cell-cycle behaviors of a population of cells generated by time-lapse microscopy. Kan Jiang et al. [45] combined two techniques for white blood cells (WBCs) segmentation. Two components of WBCs, nucleus and cytoplasm, are extracted respectively using different methods. First, a sub-image containing WBCs is separated from the cell image. Then, scale-space filtering is used to extract the nucleus region from the sub-image. Later, watershed clustering in a 3-D HSV (hue, saturation, value) histogram is processed to extract the cytoplasm region. Finally, morphological operations are performed to obtain the entire connective scheme successfully. Li et al. [52] developed a new method for WBCs identification. The method consists of the combination of an acousto-optic tunable filter (AOTF) adapter and a microscope for the image acquisition and an algorithm for data treatment. The results showed the high accuracy of the system. Pan et al. [53] trained a support vector machine model to simulate the human visual neuronal system and identify leukocytes from blood and bone marrow smear images.

Farnoosh et al. [54] developed a framework that consists of an integration of several digital image processing techniques, such as active contours, the snake algorithm and Zack thresholding for white blood cells, aiming to separate the nucleus and cytoplasm. Ritter et al. [55] presented an automatic method for segmentation and border identification of all objects that do not overlap the boundary [54]. Ongun et al. [56] did segmentation by morphological preprocessing followed by the snake-balloon algorithm [54]. Jiang et al. [45] proposed a WBC segmentation scheme on color space images using feature space clustering techniques for nucleus extraction [54]. Al-Dulaimi et al. [57] developed a WBC segmentation method using edge-based geometric active contours and the forces curvature, normal direction, and vector field. Maitra et al. [58] presented an approach to automatic segmentation and counting of RBCs in microscopic blood cell images using the Hough transform [54]. Another interesting investigation was carried out by Banik and colleagues [59]. They proposed an automatic WBC nucleus segmentation method, based on the HSI (hue, saturation, intensity), the L × a × b color space, and the k-means algorithm. This increases the generalization capability and evaluation result with a higher score on quality metrics. Then, to classify the localized WBC, they proposed a new convolutional neural network (CNN) model, which is the key factor to reduce the performance dependency between the proposed nucleus segmentation and classification method. In the end, they proved that segmentation performance does not affect the accuracy of the proposed classification method. Kawaguchi et al. [60] presented an image-based analytical method for time-lapse images of RBC and plasma dynamics with automatic segmentation. This method enabled the quantification of the perturbation-induced changes of the RBC and plasma passages in individual vessels and parenchymal microcirculation.

The literature has many more methods, however, most of the techniques presented previously were based in morphological analysis or in the form and constitution of the various blood constituents. Techniques developed for blood flows are still under development because there are many ways and methods for tracking movement. A good summary of object tracking methods can be found in [61] and cell tracking can be found in Miura et al. [62].

Recently other works appeared, for example, Dobbe et al. [63] presented a method applied to the sublingual microcirculation in a healthy volunteer and in a patient during cardiac surgery. Iqbal et al. [64] developed a novel method for the detection of abnormal behavior in cells through real-time images. The method was based in pixel classification using k-means and Bayesian classification. Chang et al. [32] segmented medical images through a charged fluid model. The model is divided in two steps defined by Poisson’s equation. Measurements of functional microcirculatory geometry and velocity distributions using image techniques have been made, such as capillaroscopy, orthogonal polarized spectral and a side-stream dark field image [63]. Ashraf et al. [65] said that “cell mobility analysis is an essential process in many biology studies”, so they have focused in developing a novel algorithm to image segmentation and tracking system conjugating the advantages of topological alignments and snakes, transforming the output of the topological alignments into the input of the active contour model to begin the analysis in the cells’ boundaries and to determine cell mobility [65]. Pan et al. [66] proposed a bacterial foraging-based edge detection (BFED) algorithm for cell image segmentation. The method was compared with the other four edge detector algorithms and showed more accurate and effective results.

In the case of Möller et al. [67], a semi-automatic tracking method with minimal user interaction was proposed. The framework was based on a topology-preserving variational segmentation approach applied to normal velocity components obtained from optical flow computations. Using the advantages of the optical flow, Kirisits et al. [68] introduced variational motion estimation for images that are defined on an evolving surface. Niazi et al. [69] studied an open-source computational method of particle tracking using MATLAB (2014 b, MathWorks, Natick, MA, US). The size and velocity of the particles are acquired from the video sequences from video-microscopic systems. The images are processed by a set of filters, selected by the user, to improve the accuracy. Park et al. [70] developed a deep learning-based super-resolution ultrasound (DL-SRU) for particle tracking. The method is based on a convolutional neural network and deep ultrasound localization microscopy. The DL-SRU was able to identify the positions of the RBCs reconstruct vessel geometry. Carboni et al. [71] used fluorescence to track blood particles flowing through a microfluidic channel. The recordings of the flow were analyzed with an algorithm developed using MATLAB to evaluate the margination parameter at relevant flows. The image processing consisted of three parts: background correction, calculation of the position and size of the particles through a gradient-based method and calculation of the displacements and velocities. Varga et al. [72] trained conventional-, deep- and convolutional neural networks to segment optical coherence tomography images to identify the number of hyperreflective foci. The networks coincide in the majority of the cases with the evaluation performed by different physicians. Chen et al. [73] studied a new approach for the segmentation of erythrocyte (red blood cell) shape. The technique was called complex local phase based subjective surfaces (CLAPSS) and presented a new variation scheme of stretching factor and was embedded with complex local phase information. The processed images were acquired by differential interference contrast (DIC) microscopy.

Some methods can also be used to track particles for diagnostic or treatments. For instance, Siegmund et al. [74] tested the use of nanoparticle labeling and magnetic resonance imaging (MRI) for in vivo tracking of adipose tissue-derived stromal cells (ASC). The labeling was stable for four months. This method has the disadvantage of not being able to identify the cell since it is an indirect method. Optimization is still required to reduce the amount of nanoparticles. Müller et al. [75] investigated the transport of magnetic particles in vessels of hen’s egg models. The flow was subjected to the influence of a magnetic field in dark field reflected light and fluorescence mode. The particles were tracked by single-particle tracking (SPT). Irreversible agglomerates were visualized after stopping the magnetic field. Consequently, further studies of the interaction between cells and particles and of the particle coating are required. Also to support the diagnosis, Kucukal et al. [76] quantified the viscosity of preprocessing-free whole blood samples from the sickle cell disease patient population by using the micro-PIV technique for in vitro assessment of whole blood viscosity and RBC adhesion. More recently, Kucukal et al. [77] have been able to measure the velocity of whole blood flow in a microchannel during coagulation using a simple optical setup and processing the images using PIV and wavelet-based optical flow velocimetry. Both studies demonstrated the viability of image processing methods to obtain data with clinical relevance. Table 1 below shows the chronological progress of the studies and that, recently, the studies have been based on automatic methods with specific algorithms and particle tracking techniques.

For studies based on in vitro approaches, there are different automatic algorithms, however, most of them still under development because the results tend to overlap at high hematocrits (Hcts), and most of them are based on images that the researchers have, taking into account their aim. Therefore, to have a good method and take advantage of all its capabilities, it is ideal to develop our own algorithm for the objective that we want to achieve. In the following sections, we will discuss the application of two automatic methods.

## 3. ImageJ Manual Plugins

ImageJ is a public domain Java image processing program. It can display, edit, analyze, process, save and print 8-bit, 16-bit, and 32-bit images. It can read many image formats including TIFF, GIF, JPEG, BMP, DICOM, FITS and “raw” data and supports “stacks”, a series of images that share a single window. It is multithreaded, so time-consuming operations such as image file reading can be performed in parallel with other operations [78]. With ImageJ [78], it is possible to calculate the area and pixel value statistics of user-defined selections. It can measure distances and angles and create density histograms and line profile plots. Moreover, it supports standard image processing functions such as contrast manipulation, sharpening, smoothing, edge detection, and median filtering [78].

**Figure 1:**
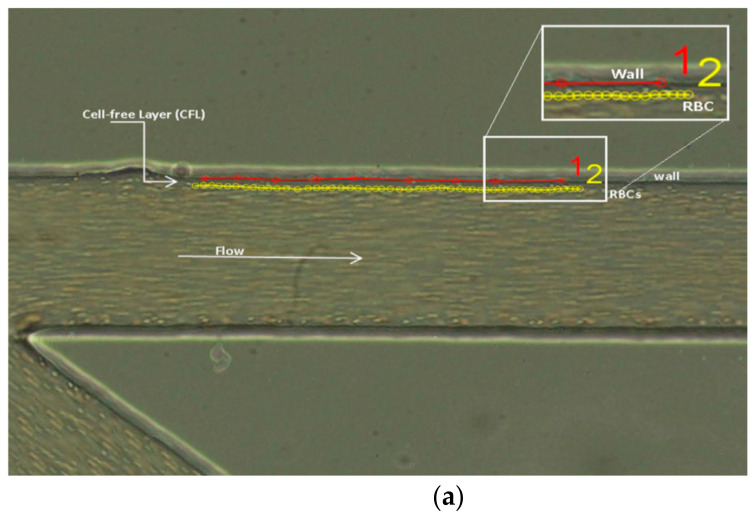

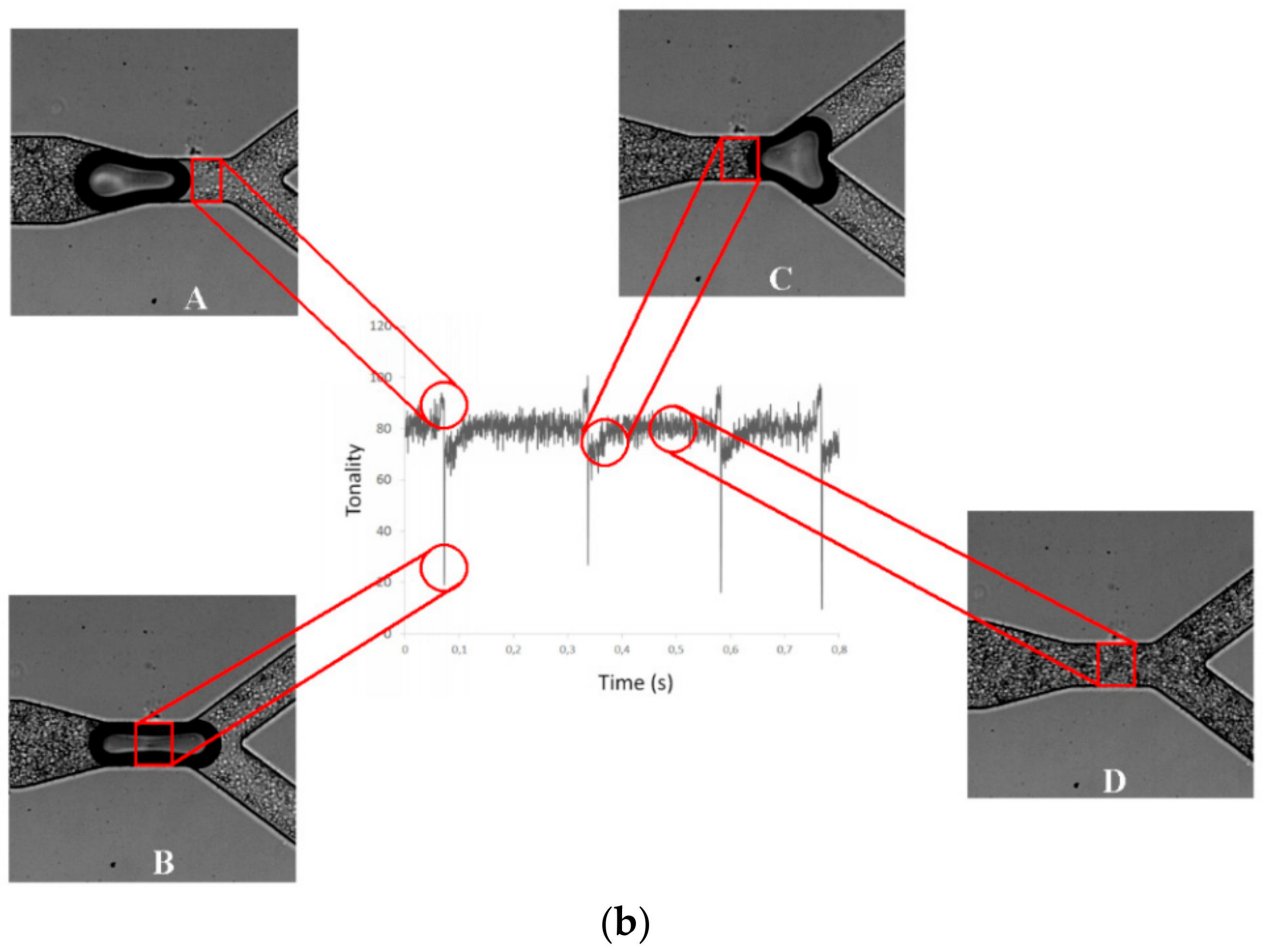
ImageJ plugins: (**a**) MtrackJ used to obtain the RBC trajectory [79] and (**b**) application of the *plot Z-axis profile* function at the selected ROI [80].

There are also different plugins to track RBCs, to count, or to measure the CFL thickness such as MtrackJ or ZProject. For example, in the study of the RBCs or other blood cell tracking, the plugin MtrackJ [49] is often used, facilitating the manual tracking of moving objects in image sequences and the measurement of basic track statistics. Through the MtrackJ plugin, the centroid of individual RBCs can be tracked, allowing obtaining the trajectory of each RBC. Additionally, it can be used to estimate RBC velocity, taking into consideration the x and y positions at each point (Figure 1a). To study the phenomena of CFL, manual tracking by MtrackJ can also be used or, as an alternative, the automatic function ZProject in ImageJ can be applied to process several images at once, creating a stack, and allowing observing the path of RBCs in the channel (Figure 1b). In Figure 1 is possible to see the application of the MtrackJ plugin to determine the CFL thickness in blood flow study [79].

**Figure 2:**
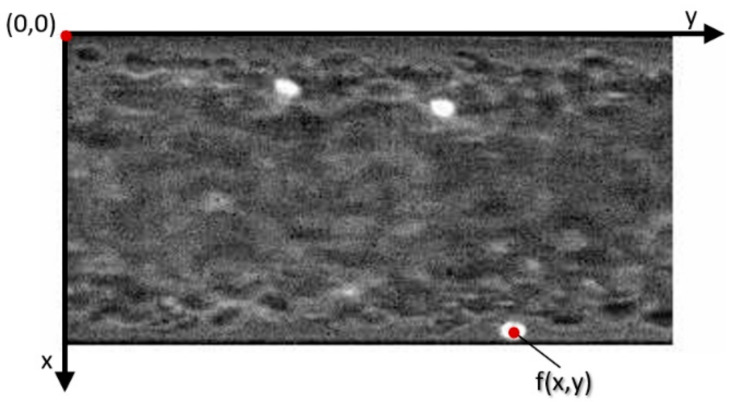
Image of blood flow in the microchannel with labeled bright RBCs, f(x,y) and the centroid of the tracking cell.

Another tool from ImageJ used in studies of blood flow is the *Plot Z-axis profile*. This function allows determining the tonality of the pixels in a region of the interest (ROI) through time. After selecting a particular area of the video the *Plot Z-axis profile* tool measures the average of tonality of the pixels in the ROI and this tonality was used as a proxy of the local hematocrit. High tonality corresponds to low hematocrit and low tonality corresponds to high hematocrit [80]. Figure 2 represents the variation in the tonality in the ROI, and consequently the variation of the hematocrit in that region, over time. 

Note that in MATLAB [27,39] there are some algorithms that researchers provide and also an application to work with ImageJ. A promising particle tracking velocimetry (PTV) plug-in for Image J is the “Particle tracker 2D and 3D” [81,82].

## 4. Automatic Image Analysis Methods

### 4.1. Red blood Cells Trajectory in a Glass Capillary

#### 4.1.1. Set-Up and Working Fluids

The confocal system used in this study consists of an inverted microscope (IX71; Olympus, Tokyo, Japan) combined with a confocal scanning unit (CSU22; Yokogawa Tokyo, Japan), a diode-pumped solid-state (DPSS) laser (Laser Quantum, Stockport, UK) with an excitation wavelength of 532 nm and a high-speed camera (Phantom v7.1; Vision Research, Wayne, NJ, USA). The laser beam was illuminated from below the microscope stage through a dry 40x objective lens with a numerical aperture (NA) equal to 0.9.

The light emitted from the fluorescent flowing RBCs, passes through a color filter into the scanning unit CSU22, where, by means of a dichromatic mirror, the light is reflected onto a high-speed camera to record the confocal images. The physiological fluid used was a solution of Dextran 40 (Dx40) with a Hct of 12%. Such was selected to obtain images with the best possible quality and consequently to reduce errors during cell tracking.

The RBCs were fluorescently labeled with a lipophilic carbocyanine derivative dye, chloromethylbenzamido (CM-Dil, C-7000, Molecular Probes, Eugene, OR, USA) using a procedure previously described [1,83]. This dye was well retained by the RBCs and had a strong light intensity, which allowed good visualization and tracking of labeled RBCs flowing in concentrated suspensions.

The microchannel used in this study was a 100 µm circular borosilicate glass capillary fabricated by Vitrocom (Mountain Lakes, NJ, USA). The capillary was mounted on a sliding glass with a thickness of 170 ± 20 µm and was immersed in glycerin to minimize the refraction from the walls.

#### 4.1.2. Manual Method

All confocal images were captured around the middle of the capillary with a resolution of 640 × 480 pixels, at a rate of 100 frames/second and then transferred to a computer for evaluation using Phantom camera control software (PH607). The manual method to track individual RBCs relies on the manual tracking plugin MTrackJ [84]. The bright centroid of the selected RBC was manually computed through successive images. After obtaining x and y positions, the data were exported for the determination of each individual RBC trajectory.

The output of this process is:

- x [µm]: The calibrated x coordinate of the point. The pixel width and unit of length used here can be set as described above.
- y [µm]: The calibrated y coordinate of the point. The pixel height and unit of length used here can be set as described above.

Figure 2 is an example of the blood flow image acquired with labeled bright RBCs and x-y coordinates.

#### 4.1.3. Automatic Method

A graphical user interface (GUI) in MATLAB was developed, for a better work environment for all users. This application must detect and track all objects that are present in a video sequence.

The algorithm is based on the steps as follows:

- Preprocessing is executed in order to remove noise and correct the brightness, and to enhance specific features of the image for increasing the robustness of the tracking procedure;
- A level of threshold is applied, in which it is possible to divide the image into different parts. The result is a binary image with a clear division between the background and objects of interest;
- The extraction procedure is done to obtain the objects’ characteristics necessary for the study.

**Figure 3:**
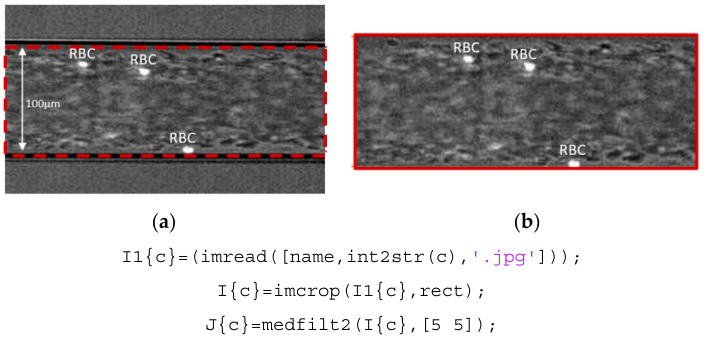
Image sequences imported (**a**) and respective region of interest cropped (**b**).

Firstly, the sequences of images were loaded to the GUI. Then, the region of interest (defined by the user) was cropped from the original images with the function *imcrop*; also a standard region is defined, but the user can change it for a better purpose. With this operation, we can work only with the region which needs to be analyzed (the region between the microchannel walls), making it easier to handle the images for the next steps, as presented in Figure 3.

**Figure 4:**
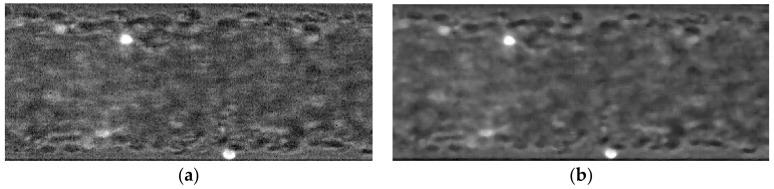
The region of interest (**a**) and the image filtered by using the median function *medfilt2* (**b**).

The next operation is the image noise elimination by applying the median filter, *medfilt2*, with one 5 × 5 pixel mask. With that, the background of the images was smooth, and the objects are enhanced preserving the edges. Figure 4 presents the result of these processes.

**Figure 5:**
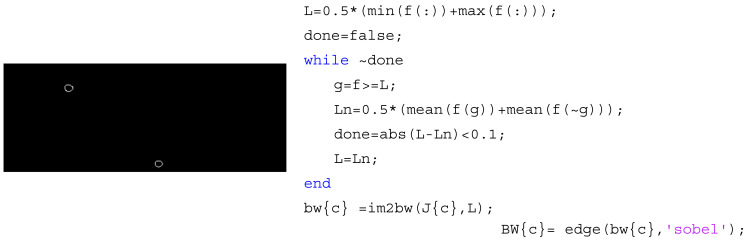
The obtained image of the iterative threshold method and the application of the Sobel filter.

In the next stage, the images were subject to a segmentation step using a threshold method. The definition of one or more values of separation is enough to divide the image into one or more regions, that is, differentiate the area of interest (the RBCs) from the not-interest area (background image). The level of threshold is calculated by default, by an iterative method, which means that for each image an adequate level of threshold is calculated. However, users can apply the value that they think to be more appropriate. After thresholding, the objects were defined with the Sobel filter (see Figure 5), which shows only the edge of the objects. The *Sobel* computes an approximation of the gradient of the image intensity. At each pixel point in the image, the result of the Sobel operator is either the corresponding gradient vector or the norm of this vector [31].

**Figure 6:**
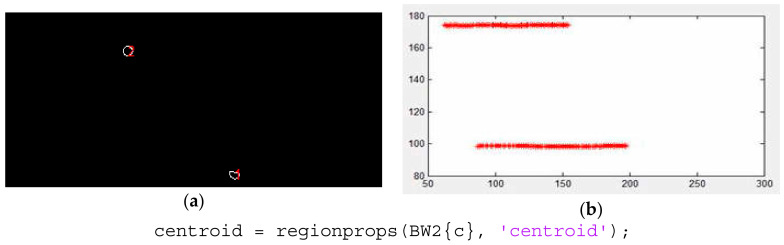
(**a**) Data extraction and (**b**) RBCs trajectories.

After the segmentation processing, the RBCs were tracked and sets of data (and positions) were obtained with the MATLAB function from the image processing toolbox, *regionprops* [27] (cf. Figure 6). This function measures a set of properties (area, centroid, etc.) for each connected component (RBC) in the binary image.

The data obtained were filtered because some of the objects are not RBC (that is, white blood cells or platelets that have higher or lower, respectively, area than the RBCs). Therefore, it is possible to filter the data by area, by imposing a minimum and maximum value. Another filter applied was the number of images where the objects are visible, because if the object has only a tracking with 10 positions, this data is not enough to be analyzed. The data with an extremely low number of tracking positions per object was eliminated.

**Figure 7:**
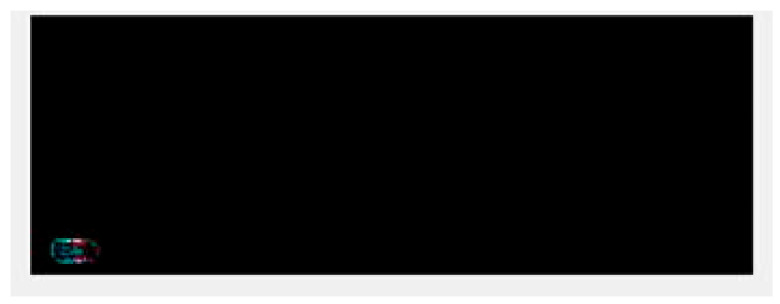
The obtained image when the Lucas Kanade pyramidal method was applied.

Another approach for this type of application is underway, which is based on optical flow. Optical flow is a technique used in computer vision algorithms to measure the speed of the pixels based on comparisons of frames, creating a field that describes the displacement that occurred between two consecutive frames of a video sequence. In other words, the optical flow consists of a dense field of velocity where each pixel in the image plane is associated with a single velocity vector [85,86]. The Kalman method and the Lucas Kanade pyramidal method were applied to the same sequence of images (cf. Figure 7).

The Lucas Kanade pyramidal method shows a better approach to the objective, but the real dimension of the object and a continuous track along the image sequence are still under development. There is a great potential in this technique to follow moving objects, such as the RBCs flowing through a glass capillary, however, due to the complexity of the method and the need for multiple variables, further investigation is required.

#### 4.1.4. Results

**Figure 8:**
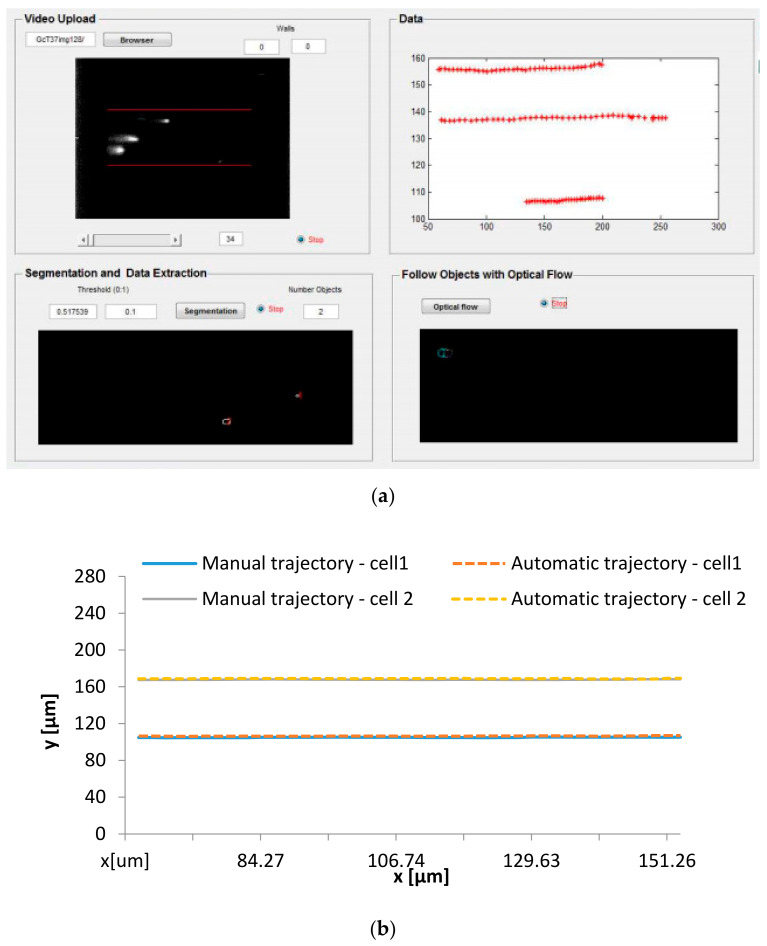
Automatic method results: (**a**) developed graphical user interface (GUI) in MATLAB and (**b**) trajectories of individual labeled RBCs determined by the manual and automatic method.

Figure 8 shows the developed graphical user interface (GUI) in MATLAB performing the image processing described in the upper sections and the trajectories of individual labeled RBCs flowing in the center plane of a microchannel, determined by the manual tracking and the proposed automatic tracking method.

The present study indicates that the data obtained from the proposed automatic method significantly matches the data obtained from the manual method. This data, x-y positions, can be used to calculate the means square deviation (MSD) and the radial dispersion (Dyy) to analyze the behavior of the RBC through a microchannel. 

### 4.2. Cell-Free Layer Thickness in a Bifurcation and Confluence Microchannel

#### 4.2.1. Set-Up and Working Fluids

The series of x-y images were captured with a resolution of 600 × 800 pixels. All images were recorded at the center plane of the microchannels at a rate of 200 frames/second, transferred to the computer and then evaluated by using an image analysis software. The microscope system used in the present study consisted of an inverted microscope (IX71, Olympus, Tokyo, Japan) combined with a high-speed camera (i-SPEED LT, Olympus, Tokyo, Japan). The blood samples used were collected from a healthy adult sheep, and ethylenediaminetetraacetic acid (EDTA) was added to prevent coagulation. The RBCs were separated from the blood by centrifugation and washed twice with physiological saline. The washed RBCs were suspended in Dextran 40 to make up the required RBCs concentration by volume.

#### 4.2.2. Manual Methods 

**Figure 9:**
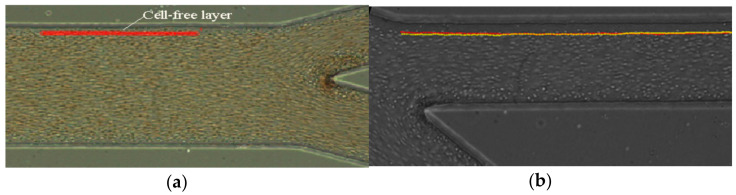
Manual method showing the trajectories of RBC defining the region of the CFL: (**a**) for an expansion geometry and (**b**) for a bifurcation geometry.

The MTrackJ plugin was used to automatically compute the centroid of the selected RBC. After obtaining x and y positions, the data was exported for the determination of each individual RBC trajectory (cf. Figure 9).

A semi-automatic method was also applied based on the use of the ZProject plugin [78]. This plugin projects an image stack along the axis perpendicular to the image plane (the so-called “z” axis) and has six different projection types.

**Figure 10:**
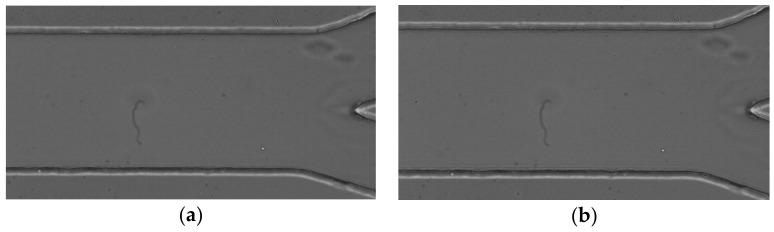
(**a**) The obtained image by applying the projection average intensity and (**b**) the obtained image by applying the projection sum slices.

**Figure 11:**
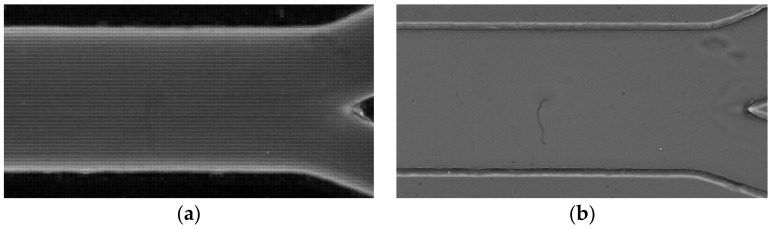
(**a**) Image obtained by applying the standard deviation projection and (**b**) image obtained by applying the median projection.

**Figure 12:**
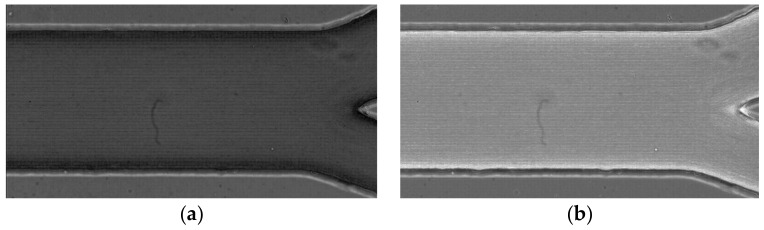
(**a**) The obtained image with the projection minimum intensity, and (**b**) the obtained image with the projection maximum intensity.

- **Average intensity projection** outputs an image where each pixel stores the average intensity over all images in the stack at the corresponding pixel location (cf. Figure 10a);
- **Sum Slices** creates a real image that is the sum of the slices in the stack (Figure 10b).
- **Standard Deviation** creates a real image containing the standard deviation of the slices (cf. Figure 11a);
- **Median** creates an image containing the median value of the slices (cf. Figure 11b).
- **Minimum intensity projection (Min)** creates an output image where each of the pixels contains the minimum value over all images in the stack at the particular pixel location (cf. Figure 12a).
- **Maximum intensity projection (Max)** creates an output image where each of the pixels contains the maximum value over all images in the stack at the particular pixel location (cf. Figure 12b).

**Figure 13:**
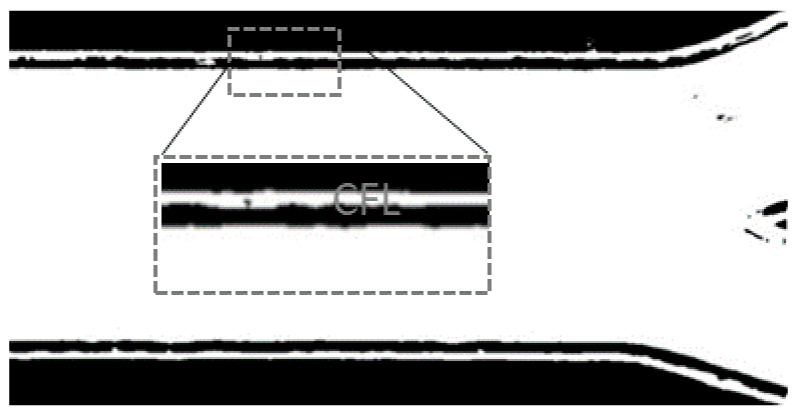
The obtained image from the ZProject method with the projection maximum intensity to extract the data. It shows a well defined CFL thickness.

After applying an appropriate projection to a stack, the resulting image is obtained, and it is then converted to a binary image (see Figure 13). The thresholding in ImageJ can be done automatically or by applying the level that the user requires.

(1) $$threshold\;=\;\frac{\left(average\;background\;+\;average\;objects\right)}{2}$$

This method works well for a good image quality and for simple geometry of the channels and represents the data accurately. Nevertheless, for more complex image data the method has some difficulties to get the correct data, so it will be necessary to specifically develop a method able to represent the data accurately.

To obtain the data, the tool *Wand* is used, which creates a selection by tracing objects of uniform color or thresholded objects. To trace an object with the *Wand* tool, it is necessary to click inside near the right edge, or outside to the left of the object. Once it finds the edge, it follows it until it returns to the starting point. The *Wand* takes the pixel value where you click as an initial value. Then, it selects a contiguous area under the condition that all pixel values in that area must be in the range initial value—tolerance to initial value + tolerance. Then the selected area will be analyzed to measure the CFL thickness.

#### 4.2.3. Automatic Method

**Figure 14:**
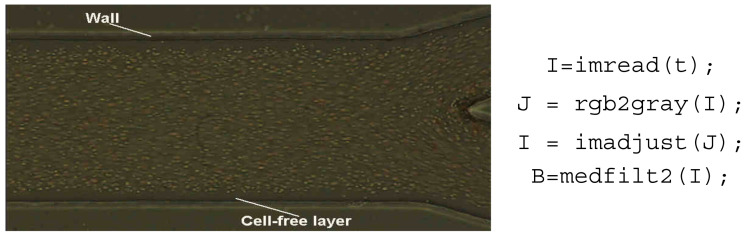
An image from the original sequence of images.

The method is based on the binarization of the sequence image. The general steps of the method are:

- Preprocessing to smooth the image and eliminate the artifacts;
- Evaluation of the intensity of all image sequences;
- Apply the binarization to the resulting image;
- Select the area to obtain the required data;

All image sequences were processed using the image processing toolbox available in MATLAB [45]. The sequence of images was loaded (cf. Figure 14), and a median filter with a 3 × 3 pixel mask was applied to each frame to reduce the noise of the images.

**Figure 15:**
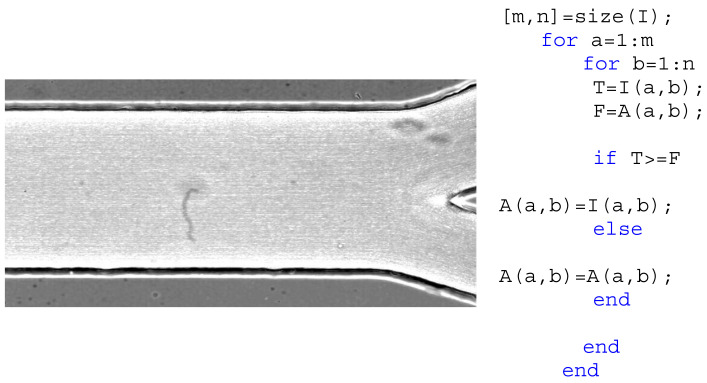
Image with the maximum intensity evaluation.

Then, the intensity of each pixel in the frame sequence was evaluated to obtain an image with the maximum intensity. With this step, it was possible to identify the region with the highest concentration of blood cells and the region where blood cells do not exist, the cell-free layer (CFL). The regions that represent the CFL have the highest intensity (white) near the microchannel walls (cf. Figure 15).

**Figure 16:**
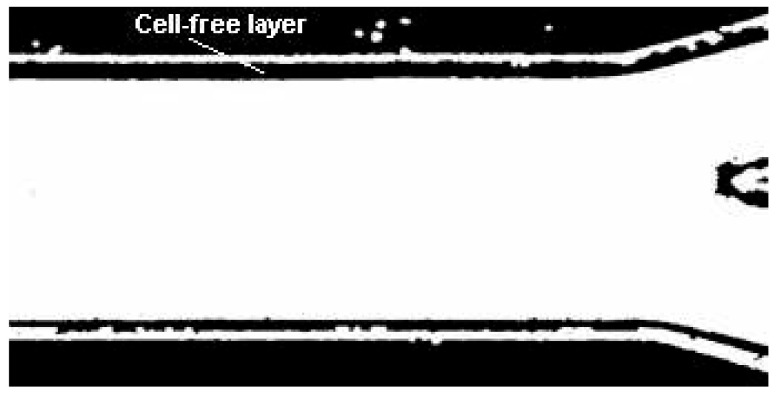
The obtained image from the automatic method.

As a final step, the image was converted into a binary image, the regions of interest were selected and the upper CFL trajectories were automatically measured. Figure 16 shows the image processing result for the developed method.

The area to take the data is defined by the user selecting the wall of the channel and the limit area from the cell-free layer.

#### 4.2.4. Results

**Figure 17:**
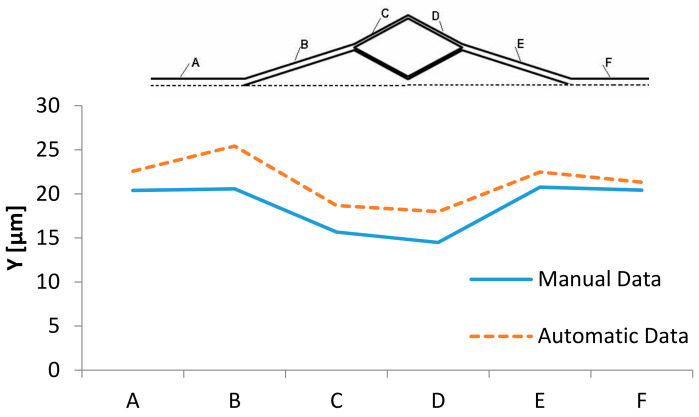
Comparison between the manual and the automatic data, taken in the regions A to F.

Figure 17 shows the results obtained by the manual method using the MtrackJ plugin and the automatic method presented in this work to measure the CFL thickness. A microchannel with bifurcation and confluence shown in Figure 15 was used for the measurements. The values obtained with both methods can be seen also in Figure 15. Data was taken in the regions represented by A to F.

It is possible to note that the data obtained by the automatic method have similar behavior with the manual data. However, the values have some discrepancies. The quality of the image and also the level of the threshold can influence this type of measurements.

## 5. Conclusions and Future Work

The present work presents not only a review on blood cells tracking methods but also comparisons of a manual method and an automatic method for two different blood flow studies. Regarding the study where RBCs were tracked through a 100 μm glass capillary, the automatic method based on a threshold algorithm was used to provide an accurate and automated process to track and as a result, it measured the RBCs flowing in microchannels. The automatic results were in good agreement with the manual method. Further work aims to implement an image analysis application able to track flowing RBCs and, consequently, extract multiple features of the RBCs that can be used in other applications, such as measuring the RBC deformability. Another method based on optical flow was also tested but it is still under development, so that it can be further improved in the future for data collection.

To study the CFL phenomenon in microchannels, the method developed based in the binarization of the image with the maximum intensity evaluation presents some discrepant results when compared to the manual data. Nonetheless, a similar qualitative tendency was observed. In this type of study, the quality of the image sequence plays a crucial role. Hence, by acquiring a sequence of images with higher quality and resolution, we believe that this automatic method can be improved and as a result, it will be able to obtain more accurate results, which should be closer to the ones obtained manually.

## Figures and Tables

**Table 1:** Summary of image analysis methods used for cell tracking and segmentation.

Reference, Year	Goal	Technical	Conclusion
[45], 2003	White blood cell (WBC) segmentation	Scale-space filtering and watershed clustering	Extracts the WBC region; The HSV space is better than the RGB space due to its low correlation.
[47], 2007	Color image segmentation	Using RGB space as the standard processing space: (1) Non-exclusive RGB segmentation. (2) Exclusive RGB segmentation.	Color images provide a better description of a scene as compared to grayscale images
[54], 2009	WBC segmentation: to separate the nucleus and cytoplasm	It is based on the morphological analysis and the pixel intensity threshold, respectively.	The method is able to yield 92% accuracy for nucleus segmentation and 78% for cytoplasm segmentation.
[60], 2012	To quantify the perturbation-induced changes of the RBC and plasma passages in the individual vessels.	The image-based analytical method for time-lapse images of RBC and plasma dynamics with automatic segmentation	Arterial tones and parenchymal blood flow can be individually coordinated.
[52], 2013	To segment the nuclei and cytoplasm of WBCs	It is based on the pixel-wise ISAM segmentation algorithm	the accuracy of the proposed algorithm is 91.06% (nuclei) and 85.59% (cytoplasm)
[67], 2014	Cell tracking	Topology preservation techniques	The method has good accuracy
[71], 2016	Direct particle tracking	Algorithm developed in MATLAB	Results obtained confirm experimental results
[66], 2017	Optimize traditional edge detection	Edge detection algorithm based on bacterial liner	Identifies boundaries more effectively and provides more accurate image segmentation
[69], 2019	Determine particle velocity and size distributions of large groups of particles by video-microscopic systems.	Open-source computational implementation with MATLAB	It allows the automatic tracking of any fluid with particles, classifies the particles according to their size and calculates the speed.
[70], 2020	Particle tracking	The method is based on a convolutional neural network and deep ultrasound localization microscopy	Its robust, fast and accurate RBC localization, compared with other ULM techniques
[76], 2020	In vitro assessment of whole blood viscosity (WBV) and RBC adhesion	Micro-PIV	WBV and RBC adhesion may serve as clinically relevant biomarkers and endpoints in assessing emerging targeted and curative therapies in SCD.
[77], 2021	Measurements of the velocity of whole blood flow in a microchannel during coagulation	PIV and wavelet-based optical flow velocimetry (wOFV)	The high-resolution wOFV results yield highly detailed information regarding thrombus formation and corresponding flow evolution
